# The Potential Role of Social Media Platforms in Community Awareness of Antibiotic Use in the Gulf Cooperation Council States: Luxury or Necessity?

**DOI:** 10.2196/jmir.3891

**Published:** 2015-10-15

**Authors:** Hosam Mamoon Zowawi, Malak Abedalthagafi, Florie A Mar, Turki Almalki, Abdullah H Kutbi, Tiffany Harris-Brown, Stephan Harbarth, Hanan H Balkhy, David L Paterson, Rihab Abdalazez Hasanain

**Affiliations:** ^1^ The University of Queensland, UQ Centre for Clinical Research Herston QLD 4029 Australia; ^2^ King Saud bin Abdulaziz University for Health Sciences College of Medicine Riyadh Saudi Arabia; ^3^ WHO Collaborating Centre for Infection Prevention and Control and GCC Center for Infection Control Riyadh Saudi Arabia; ^4^ Department of Pathology, Brigham and Women’s Hospital, Harvard Medical School Boston, MA 02215, MA United States; ^5^ Department of Pathology, University of California, San Francisco CA 94143, USA, CA United States; ^6^ Faculty of Medicine Taif University Taif Saudi Arabia; ^7^ Infection Control Programme and WHO Collaborating Centre on Patient Safety University Hospitals of Geneva Rue Gabrielle-Perret-Gentil 4, Switzerland; ^8^ School of Public Health Queensland University of Technology QLD Australia

**Keywords:** social media, antibiotics, awareness, health campaigns, Gulf States

## Abstract

The increasing emergence and spread of antimicrobial resistance (AMR) is a serious public health issue. Increasing the awareness of the general public about appropriate antibiotic use is a key factor for combating this issue. Several public media campaigns worldwide have been launched; however, such campaigns can be costly and the outcomes are variable and difficult to assess. Social media platforms, including Twitter, Facebook, and YouTube, are now frequently utilized to address health-related issues. In many geographical locations, such as the countries of the Gulf Cooperation Council (GCC) States (Saudi Arabia, United Arab Emirates, Kuwait, Oman, Qatar, and Bahrain), these platforms are becoming increasingly popular. The socioeconomic status of the GCC states and their reliable communication and networking infrastructure has allowed the penetration and scalability of these platforms in the region. This might explain why the Saudi Ministry of Health is using social media platforms alongside various other media platforms in a large-scale public awareness campaign to educate at-risk communities about the recently emerged Middle East respiratory syndrome coronavirus (MERS-CoV). This paper discusses the potential for using social media tools as cost-efficient and mass education platforms to raise awareness of appropriate antibiotic use in the general public and in the medical communities of the Arabian Peninsula.

##  Introduction

Antibiotic resistance has become a severe public health threat worldwide, including in the Gulf Cooperation Council (GCC) states [1]. Hence, various initiatives across the globe have been launched to combat this issue. In 2011, the World Health Organization (WHO) themed its annual day to address this issue with the slogan “No action today, no cure tomorrow” and listed the actions to be undertaken. These actions included providing education to achieve effective antibiotic use [2]. Antibiotic misuse, such as demanding unneeded antibiotics, purchasing antibiotics over-the-counter without a prescription, and not completing a course of antibiotics, is associated with the emergence and selection of antibiotic-resistant bacteria [3]. Nosocomial infections caused by antibiotic-resistant pathogens are significantly associated with an increased length of hospital stay and increased cost [4].

European surveys have shown that the general public still misunderstands the function and correct use of antibiotics [5,6]. On many occasions, the WHO has highlighted the importance of involving the general public alongside health care professionals for combating the emergence of antimicrobial resistance (AMR) [7,8]. To alleviate the public’s lack of cognizance about AMR, various countries in Europe [7,9], as well as the United States [10] and other countries, have initiated public campaigns to raise awareness about the appropriate use of antibiotics.

Considering Saudi Arabia and other Gulf countries, various studies have addressed the issue of antibiotic misuse in hospital settings and the easy over-the-counter access to prescription antibiotics. For example, antibiotic use in intensive care units (ICUs) in Saudi Arabia has been found to be 10 times greater than that in the United States and some European countries [1,11-13]. This issue also extends to the wider GCC community. A Saudi study found that 77.6% of pharmacies dispensed antibiotics without a prescription primarily to treat scenarios consistent with viral infections [14], whereas 68.4% of antibiotics from Abu Dhabi pharmacies were sold over-the-counter [15], suggesting lack of antibiotics knowledge [16].

These examples of antibiotics misuse and others suggest the urgent need for a public campaign in Saudi Arabia and beyond to provide greater education on the proper use of antibiotics along with the concept of reserving antibiotics for use only when they are truly needed. In April 2015, the GCC Center for Infection Control released the multilevel GCC Strategic Plan for Combating Antimicrobial Resistance, which sets the framework for the regional and national plans [17]. One of the 5 strategic roadmaps addressed is the importance of preserving and restricting the available antimicrobial agents for human use. Interventional methods include educating antibiotic prescribers, patients, and the general public on the importance of appropriate antimicrobials use and basic infection prevention and control (eg, immunization and hygiene) [17].

Social media platforms are being widely used for health promotion advocates and to endorse traditional awareness campaigns. They have unique characteristics for sharing open access information, providing a platform for dynamic conversations with communities and social groups, and keeping users connected with their topics of interest [18]. They have been used to raise awareness for obesity, diabetes [19], and adolescent dating violence [20]. As an outreach effort, the European Antibiotic Awareness Day released a toolkit to advise on how to engage in social media activities promoting prudent antibiotic use [18]. Information provided in the toolkit relates to European countries and may not fully apply to the GCC states.

In this paper, we discuss the planning, setup, and potential effectiveness of developing a mass education campaign via social media platforms to raise general public and medical awareness of appropriate antibiotic usage in the GCC states.

### The Influence of Educational Campaigns on Antibiotic Use and Antibiotic Resistance

Before initiating an educational media campaign, it is important to review the effectiveness of previous initiatives that were developed and launched in other parts of the world to raise awareness and provide guidance on responsible antibiotic use (Table 1).

**Table 1:** Summary of selected antibiotic awareness campaigns worldwide.

Name	Site (country)	Duration	Method used	Target audience	Reduction rate
Belgian Antibiotic Policy Coordinating Committee (BAPCOC ) establishment [21]	Belgium	Launched in 1999-present	Multimedia campaigns, national campaigns, publication of clinical practice guidelines, support for the establishment of antibiotic management teams (AMTs)	Belgian community	36% reduction in outpatient antibiotic use per 1000 inhabitants per day during winter season
“Les antibiotiques c’est pas automatique” (“Antibiotics are not automatic”); part of the national campaign “Keep antibiotics working” [22]	All 22 regions across France	2001-2007	Mass media campaigns, one-on-one physician education sessions	General public and health care professionals	26.5% reduction of antibiotic prescriptions per 100 inhabitants during winter season over a 5-year period
Antibiotics Awareness Week [23]	Australia	2012-present	Facebook, Twitter, online pledging	All Australians	Unknown as yet
Local low-cost information campaign [9]	Emilia-Romagna region (Northern Italy)	November 2011-Februrary 2012	Brochures, posters, local media advertisements, and visual aids	General public	4.3% reduction in defined daily doses of prescribed antibiotics in intervention group
English public antibiotic campaigns [7]	England and Scotland	2008	Posters displayed in magazines and newspapers	General public	No improvement observed in postsurvey of public’s understanding
e-Bug [24]	European countries and Saudi Arabia	Launched in 2006-present	Website-based games	Junior and senior school students	Not assessed

Data from Belgium and France have also revealed a reduction in the misuse of antibiotics after educational interventions. A decrease of 26.5% in antibiotic prescriptions was observed in France between 2002 and 2007 compared with the preintervention period (2000-2002), with the largest reduction observed in children [22]. A 36% reduction in antibiotic prescriptions was also observed in Belgium from 1999-2000 to 2006-2007 [21]. Both countries also reported a decrease in the incidence of infection with invasive penicillin- and macrolide-resistant *Streptococcus pneumoniae*. It was noted that the decrease in Belgium occurred before the wider use of the pneumococcal conjugate vaccine (PCV7), indicating that the vaccine did not contribute to this initial reduction in the incidence of infection with invasive penicillin-resistant pneumococci [5,6]. These data suggest the effectiveness of antibiotic awareness media campaigns in decreasing the use of antibiotics and hence in reducing the impact of antibiotic resistance.

Recently, Formoso et al [9] reported the effectiveness of a low-cost media campaign on antibiotic use in an Italian province that lasted for 5 months during the cold season. The intervention materials included visual aids, such as posters, brochures, and advertisements, which were used in the local media. The key messages of the campaign were codesigned by a physician practicing in the intervention area. Antibiotic prescriptions were significantly reduced by 4.3% in the intervention area compared with the control area. However, the general population’s knowledge and attitudes about antibiotic resistance were not changed by the campaign [9].

In 2008, antibiotic awareness campaigns were carried out in England and Scotland by broadcasting key messages in advertising published in magazines and newspapers. Unfortunately, the campaign did not show any positive effects in either England or Scotland. No improvement was observed in the general public’s understanding of antibiotic misuse to treat coughs and colds despite the fact that in 2009 more public respondents agreed that “resistance to antibiotics is a problem in British hospitals” than in 2008 [7]. In fact, this is not the only documented failure of antibiotic awareness campaigns. Huttner et al [5] reviewed 22 campaigns launched in high-income countries between 1990 and 2007. At least 3 of these campaigns failed and the effect of 3 others is unknown because of a lack of follow-up assessment on antibiotic use [5].

The e-Bug project is an example of an innovative approach to raise awareness about microbes and infection prevention. The aim of the e-Bug project is to disseminate educational materials about microbes (both beneficial and pathogenic) to junior and senior school students across Europe. The project relies on website-based gaming and entertainment-based lessons [24]. The e-Bug project now has partners in 26 different European countries, as well as Saudi Arabia, providing educational materials in different European languages and in Arabic [25]. On May 2015, the e-Bug website had a total of 17,391 visitors and the Saudi Arabian site had 76 visitors in total [26]. The impact of the e-Bug project is not clear because no evaluation of its implementation and impact on behavioral changes in targeted groups was ever performed [24].

Various factors have been suggested to be necessary to achieve success in antibiotic awareness campaigns. These include carefully designing key messages that are clear and simple, targeting both general public individuals and clinicians, and using television and radio [6,7]. Moreover, motivating physicians to be involved in communicating with patients about the appropriate use of antibiotics and antibiotic resistance is also important [7]. Physicians’ participation in developing campaign messages and communicating with the general public might significantly improve the chances of success. Early engagement can also have an impact on the sense of ownership of the campaign and facilitate physicians’ consistent support [27]. This may indirectly influence physicians, focusing their attention on antibiotic prescribing and providing greater patient education.

### Variable Sociological Factors

Awareness messages that display local surveillance data or amount of antibiotics locally misused may be important. Sharing with the general public the real-life experiences of individuals who have been infected with “superbugs” could be useful and may help the audience identify with those affected. Sharing real-life medical experience has been shown to be a useful communication platform from which to clarify public health-related stories, such as acne and cancer [28,29]. Considering these factors when designing awareness campaigns about antibiotics may help make an impact in GCC communities. Based on the Health Belief Model, perceived susceptibility (ie, you are at risk of getting infected) can be used to raise awareness [30,31]. However, delivering known messages to the target audience might result in a loss of interest and later disengagement. Educational interventions would be more successful if local contexts and barriers are adequately analyzed and addressed.

Replicating campaign strategies that have been initiated in different global regions outside the GCC region might not result in an effective outcome. It is crucially important to study the cultural factors and antibiotic distribution infrastructure in the GCC before thinking about the awareness messages. For example, the United States’ Get Smart campaign [32] highly recommends that parents do not demand antibiotics for their children from the treating physician. We do not believe that this message line will be as effective as it might be in the United States because antibiotics can be purchased without a prescription from community pharmacies; therefore, a doctor’s refusal might not make a difference. Approximately 37% of the total population of the GCC states consists of nonnational expatriates [33]; hence, it is important to consider cultural differences and not to neglect this segment when setting up a public awareness campaign. For example, the Saudi Ministry of Health has generated educational materials in multiple languages to fulfill this requirement [34].

Community pharmacies have a significant role in dispensing antibiotics in GCC communities. For example, 24.4% of 1645 recently surveyed antibiotic transactions in community pharmacies in Abu Dhabi were carried out without a prescription, including amoxicillin-clavulanic acid for sore throats and ceftriaxone for sexually transmitted infections [35]. The illegal practice of selling antibiotics over-the-counter, without a prescription, did not favor expatriates over citizens in the surveyed pharmacies in Abu Dhabi [15].

The self-prescription of antibiotics is another sociological factor that must be considered when designing antibiotic awareness campaigns in the GCC states [36,37]. This factor is strongly associated with the availability of antibiotics over-the-counter.

Before creating content to be used for awareness campaigns, it is necessary to conduct formative research to assess the public’s existing knowledge of antibiotics resistance, understand the motivations for inappropriate antibiotics use, and learn about the social and cultural backgrounds for the targeted population. That will subsequently help to develop tailored key messages that can potentially encourage behavioral change [30]. Knowing these critical elements has lead to the success of many awareness campaigns, such as The Magic Glasses video to prevent soil-transmitted helminthes in China [31,38]. On the other hand, content produced for social media-based campaigns can be unrelated to the campaign’s target. For example, it was found that the majority of Movember campaign-related tweets did not associate with prostatic and testicular cancer awareness [39], and the majority of tweets produced during breast cancer awareness week did not promote any specific preventive behaviors [40]. Despite the importance of developing related content, research into the correlation between social media-based awareness campaigns and behavior change is minimal because it is a new avenue in public health awareness [41].

Social media platforms can also contain contradictory health messages with potentially negative impact. Because social media platforms give users the freedom to publish their content, some of that content can contain medically misleading information, as found in YouTube videos promoting anorexia [42].

### Funding

Funding is an important factor that may significantly affect a campaign’s functionality and outcome. A systematic review of more than 20 international campaigns aimed at raising awareness of antibiotic use showed that these campaigns sourced their funding from different sectors, including the pharmaceutical industry [6]. The funding spent by pharmaceutical companies on promoting and marketing antibiotics is massive. For example, in 1998, it was estimated that pharmaceutical companies in the United States spent approximately US $1.6 billion to promote antibiotics [43]. On the other hand, media campaigns that encourage the prudent use of antibiotics are not widely supported [6]. Government funding is important. Because antibiotic awareness campaigns might translate into wiser use of antibiotics and potentially lead to a reduced selection of resistant bacteria, public funding should be offered to support awareness campaigns.

The cost of running a traditional mass media campaign to promote prudent antibiotic use in the community can be very expensive. For example, developing and conducting the French antibiotic awareness campaign carried out from 2002 to 2007 cost approximately €500 million over a 6-year period [44], whereas the Belgian campaign cost considerably less at approximately €400,000 per year [21]. The “Get Smart Colorado” campaign, which took place for 4 months in 2002, reported a cost of US $88,500 to purchase advertising space that included bus tails, bus stop posters, interior bus signs, and national public radio spots [10]. Similarly, the recent Italian campaign in 2013 cost approximately US $60,800 for purchasing media spots on television and radio, and in newspapers. Approximately the same cost was spent to develop and print written visual aid materials, such as posters [9].

Considering social media platforms are free, establishing a social media-based campaign may be far cheaper than traditional media-based campaigns. However, in order to maintain continuous cyber presence and followers scalability, social media managers are usually hired [45], which can be an additional cost burden on social media-based campaigns. The key advantage of social media is the possibility to measure and track impressions and responses to online posts. These data can be used to guide social media campaigns to improve marketing strategy. However, platforms available to analyze big data generated from social media can be costly and may require technical expertise.

### Time

Repeating the educational intervention over a long period of time is essential for the awareness success of mass media campaigns. Repetition over a long period of time has been demonstrated for causes such as smoking cessation and has helped achieve effectiveness [46]. The vast majority of antibiotic awareness campaigns launched in high-income countries between 1990 and 2007 were conducted over a period of more than 1 year [6]. Other campaigns, such as European Antibiotic Awareness Day (on November 18 each year) [47], are seasonal and have a long-term sustainable plan. However, the “Get Smart Colorado” campaign, which lasted for only 4 months, successfully showed a 3.8% net reduction in antibiotic dispensing at retail pharmacies as well as an 8.8% net decrease in managed care-associated antibiotic dispensing [10].

The ease of using social media, along with the indirect community contribution via “share” and “retweet” features, might provide long-term exposure and awareness messages to the wider general public. However, it is important to consider the temporal effect of social media feeds due to their short lifespan. It was found that the half-life of a tweet is approximately 24 minutes, whereas the half-life of Facebook posts is approximately 90 minutes [48]. A hashtag is a keyword preceded by a hash sign (#) that is used to identify and categorize messages on a specific topic, which can give the topic a longer lifespan in social media [49,50]. Keeping the audience engaged and interested in the topic is another important consideration. This might be achieved by ensuring that the key messages and materials are not overrepeated throughout the campaign’s duration. Updating campaign materials with new and relevant data might keep the audience more engaged and keen to receive updated educational materials. Lastly, the time chosen to post the social media message can be critical for the lifespan of social media posts [51].

### The Value of Social Media Platforms to Communities in the Gulf Cooperation Council States

The total number of users of the social networking website Facebook in the Arab world (22 countries) had grown to 54,552,875 by the end of May 2013; 33.4% of users are female and 68% are younger than 30 years [52]. Facebook users in the GCC states represent approximately 22% (12 million) of the total Facebook users in the Arab world [52] (Table 2).

**Table 2:** The use of Internet and social media platforms in the GCC states.

Country	Population (million)^a^	Internet users (million), n (%)^b^	Facebook users (million),^c^ n (%)	Twitter users (million),^d^ n (%)
Saudi Arabia	28.4	13.0 (45.8)	6.4 (22.5)	1.9 (6.7)
United Arab Emirates	8.3	5.7 (71.0)	3.4 (41.7)	0.4 (4.8)
Kuwait	3.1	2.0 (63.2)	0.8 (26.8)	0.2 (7.3)
Bahrain	1.2	1.0 (80.0)	0.3 (25.1)	0.1 (5.6)
Qatar	1.7	1.7 (99.9)	0.6 (34.4)	0.1 (4.4)
Oman	3.3	2.1 (63.6)	0.5 (16.4)	0.04 (1.2)

^a^ Population figures obtained from [53].

^b^ Internet user figures obtained from [54].

^c^ Facebook user figures obtained from [52].

^d^ Twitter user figures obtained from [55].

For the microblogging website Twitter, the number of active users in the Arab world reached 3,766,160 individuals as of March 2013, with an estimated 10,832,000 tweets per day. Saudi Arabia has the highest number of active Twitter users in the Arab world, with 1.9 million individuals, which is approximately 50% of the total Twitter users in the Arab region. Approximately 47% and 11% of the total tweets in the Arab world are generated from Saudi Arabia and United Arab Emirates, respectively.

The video-sharing website YouTube is also a popular media platform in the GCC, particularly in Saudi Arabia. As an update to research conducted by Forbes Middle East, we present data from selected local GCC talk shows on YouTube (Table 3). It is clear that these shows attract many viewers, although some shows from Saudi Arabia receive the most attention. This audience would make these shows an excellent platform for delivering awareness messages to a larger number of viewers.

**Table 3:** The popularity of selected YouTube-based shows in the GCC states.

Name of show	Origin	Launch date	Episodes^a^	Subscribers^a^	Total views^a^	Average views per episode^a^
EyshElly	Saudi Arabia	Feb 2011	63	1,714,699	197,686,128	3,137,875
3al6ayer	Saudi Arabia	Sep 2010	45	810,119	61,462,338	1,365,029
	Saudi Arabia	Sep 2010	33	649,465	72,887,865	2,208,723
Endam Cinema	Oman	Jul 2013	6	215	50,864	8477
Balalee6	United Arab Emirates	Jun 2012	13	6686	972,165	74,781
	United Arab Emirates	May 2012	5	10,133	954,815	190,963
shenoya3nitv	Kuwait	Jan 2012	54	67,477	8,076,193	149,559
How to Prevent from Corona	Saudi Arabia	May 2014	1	17,759	2,973,376	NA

^a^ The figures were obtained from YouTube channels on November 15, 2013.

### Examples of Saudi Public Health Awareness Messages Delivered Through Social Media Platforms

Owing to its high profile and popularity among Internet users in the GCC region, YouTube has often been used in Saudi Arabia as a platform to deliver public health–related awareness messages and campaigns (Table 4). We have noticed 2 different models for delivering health-related topics on YouTube in Saudi Arabia. One model was noticed in many campaigns that used comedy talk shows with large audience as a platform to deliver the awareness messages. With the help of other social media platforms, such as Facebook and Twitter, these messages have traveled far and wide, attracting a large number of viewers. For example, a Saudi-based comedy show named “Fe2aFala” released an episode about acquired immune deficiency syndrome (AIDS) and this episode attracted over 1 million viewers.

**Table 4:** Health messages delivered through Saudi YouTube-based shows.^a^

Show/channel name	Type	Awareness about	Number of viewers	Channel subscribers
Phosphine	Special episode	Phosphine gas	3,288,241^b^	14,821
Lumink	Special show	Health promotion	2,583,534	807,561
Telfaz11	Special episode	Breast cancer	1,489,802	296,563
Fe2aFala	Special episode	AIDS	1,139,948	351,907
Sen_tube	Entire show	Dental care	1,292,959	29,373
3almezan	Entire show	Obesity and well-being	1,097,713	51,746
Hotcoldshow	Special episode	Diabetes	455,485	81,725
MedScoope	Entire show	Health promotion	88,703	937

^a^ The figures were obtained by accessing the YouTube channels on the March 4, 2014.

^b^ This number of views was achieved within only 3 days of uploading the video on YouTube.

Another model is to use YouTube as a channel similar to traditional mass media to distribute health awareness messages. For example, in June 2014, the Saudi Arabia Ministry of Health launched an engaging public awareness campaign using YouTube, Twitter, Facebook, educational posters, and health guideline updates to educate the general public and medical communities on the emergence of, and health precautions needed for, MERS-CoV [34]. In this example, social media platforms may have been used to create online presence to endorse awareness messages delivered locally on traditional media. It must be noted that the incidence rate of MERS-CoV has declined [56] with the multi-faceted intervention, which social media has been a part of.

Publishing awareness content on social media might allow international distribution and an indefinite exposure period. However, in order to potentiate the effectiveness of a YouTube video, additional advertising strategies must take place. Table 4 summarizes some of the campaigns that have used YouTube shows to deliver public health awareness in Saudi Arabia and have attracted a high number of views.

### Our Pilot Experience

Many health care facilities and organizations have started using Twitter as a teaching tool; for example, “tweeting” about specific health problems has been used by major organizations such as the Centers for Disease Control and Prevention (CDC), WHO, the National Cancer Institute (NCI), and the National Institutes of Health (NIH). “Tweets” go out from a sender and are simultaneously received by all members of a group of “followers,” providing a fast, open, and easy way to deliver a particular focused message.

We used Twitter in our online Arabic-language pilot campaign focusing on superbugs using a hashtag. We delivered short tweets and links to various articles and videos related to superbugs. We also translated multiple non-Arabic research articles and news for our more than 34,000 followers, who were primarily from Saudi Arabia and the surrounding areas. This medium gave us the ability to have a real-time conversation by answering their questions and concerns about the topic. To track our efforts, we regularly reviewed the number of followers, updates, retweets, and “mentions” in Twitter. Many evaluation metrics for Twitter can be collected. We evaluated our Twitter pilot study by analyzing the influence of some of our tweets. We kept track of how many of our “followers” published updates including “retweets” or “at replies” over time. A simple analysis of 147 selected tweets with the Arabic superbug hashtag resulted in approximately 4100 retweets between July and November 2013. This information does not tell us which of the tweets encouraged followers to go on to read the full article or watch the video, but it does give us useful information on general interest levels.

We created a whiteboard animation with a voiceover video [57] that discussed the importance of antibiotics, how superbugs are created, and the spread of superbugs in the GCC. We also discussed possible factors that could contribute to the emergence and spread of superbugs in addition to advice on how to control superbugs as reviewed previously [1].

**Figure 1:**
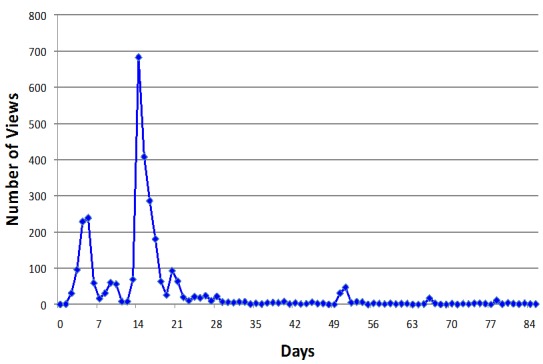
YouTube views over a period of 84 days for the whiteboard animated and voiceover video about superbugs and proper antibiotics use.

Interestingly, the number of retweets that we received from our superbugs hashtag correlated with the number of YouTube views that we recorded for our video. The video viewing pattern as determined by YouTube was as expected: there were a large number of views soon after the video was posted and released on Twitter, and there were fewer views from 3 weeks later until the present day. We observed 2 large peaks in viewing rates within the first 2 weeks of the video being released. We believe the first one was due to our successful Twitter activities. We are unclear about what caused the second and largest jump in YouTube views. It is possible that the video was picked up by another group and spread on Twitter again (Figure 1).

We also observed sporadic smaller peaks in YouTube views from 3 weeks onward. These numbers suggest that the video was still drawing attention and being shared, most likely on social media, even months after being posted.

Our viewer retention rate was consistent with what YouTube estimates to be the average retention rate for videos of a similar length—approximately half of the video. This rate suggests that the length of our video—5.5 minutes—is adequate to capture the viewers’ attention.

**Figure 2:**
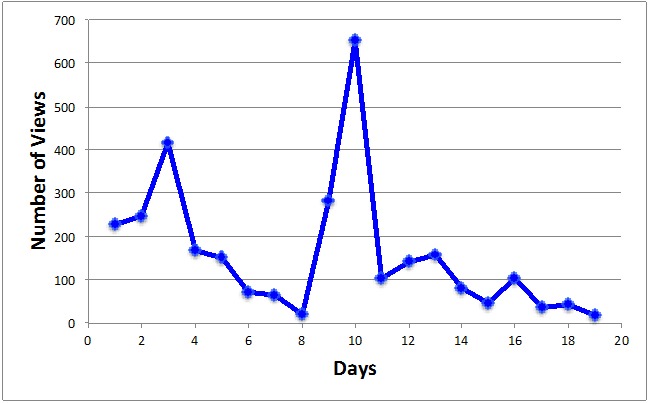
YouTube views over a period of 20 days for the infographic video.

Subsequently, we created an infographic video uploaded on YouTube [58] that combined a selection of our tweets with visual and audio enhancement. The tweet that featured the video received 152 retweets and 130 favorites, and the video was viewed more than 3000 times in only 2 weeks (Figure 2).

The content generated for our pilot campaign was primarily dependent on our existing knowledge of antibiotic misuse in Saudi Arabia and neighboring states as previously reviewed [1,14-16,35-37]. We acknowledge the limitations of our pilot campaign resulting from the lack of formative research, its Arabic content, and reliance on social media. In order to achieve prospective behavioral change, it is important to understand the motivation for antibiotic misuse in the general public. Future research should conduct a thorough survey, interviews, and observations to cover the diverse population of the GCC states. This will help us design relevant key messages to be used in future awareness campaigns. The diverse ethnic groups and socioeconomic status of people in the Gulf States should also be considered in future campaigns because more languages and media platforms might need to be used. Relying solely on a social media platform in an awareness campaign might overlook the large population of migrant workers in the GCC who might not have access to social media platforms.

### Conclusion

Reducing the suboptimal use of antibiotics among the general public and medical community through awareness activities is an important element in national plans to combat rising AMR. However, it is important to create awareness content that is related to the target audience and based on formative research. Social media platforms seem to be a valuable platform for delivering awareness messages. Owing to social media popularity, awareness messages could reach a large number of users and the reach can be tracked. Through our pilot experience we have successfully distributed antibiotics awareness messages through Twitter and YouTube to our target audience in the Gulf counties and Saudi Arabia. The use of social media can also enhance awareness campaigns delivered in traditional media channels. However, it is important to consider the cultural demographic diversity, which could limit the reach of awareness massages, such as the high population of immigrant workers in the GCC who might not have access to these emerging social media platforms. Social media–based messages can also have short life span, which might limit the effect and reach of key awareness messages. Measuring the impact of the social media–based awareness campaigns on the general public’s understanding and behavioral change is a challenge and needs further research.

## Abbreviations

AIDS: acquired immune deficiency syndrome
AMR: antimicrobial resistance
GCC: Gulf Cooperation Council
ICU: intensive care unit
NCI: National Cancer Institute
NIH: National Institutes of Health
WHO: World Health Organization
